# Brain Asymmetry and Its Effects on Gait Strategies in Hemiplegic Patients: New Rehabilitative Conceptions

**DOI:** 10.3390/brainsci12060798

**Published:** 2022-06-18

**Authors:** Luca Vismara, Veronica Cimolin, Francesca Buffone, Matteo Bigoni, Daniela Clerici, Serena Cerfoglio, Manuela Galli, Alessandro Mauro

**Affiliations:** 1 Division of Neurology and Neurorehabilitation, IRCCS Istituto Auxologico Italiano, 28824 Verbania, Italy; veronica.cimolin@polimi.it (V.C.); m.bigoni@auxologico.it (M.B.); d.clerici@auxologico.it (D.C.); serena.cerfoglio@polimi.it (S.C.); alessandro.mauro@unito.it (A.M.); 2 Department of Neurosciences “Rita Levi Montalcini”, University of Turin, 10126 Turin, Italy; 3 Department of Electronics, Information and Bioengineering, Politecnico di Milano, 20133 Milano, Italy; manuela.galli@polimi.it; 4 Principles and Practice of Clinical Research (PPCR), Harvard T.H. Chan School of Public Health–ECPE, Boston, MA 02115, USA

**Keywords:** hemiplegia, hemiparetic, ischaemic stroke, brain asymmetry, gait analysis, retrospective cohort study

## Abstract

Brain asymmetry is connected with motor performance, suggesting that hemiparetic patients have different gait patterns depending on the side of the lesion. This retrospective cohort study aims to further investigate the difference between right and left hemiplegia in order to assess whether the injured side can influence the patient’s clinical characteristics concerning gait, thus providing insights for new personalized rehabilitation strategies. The data from 33 stroke patients (17 with left and 16 with right hemiplegia) were retrospectively compared with each other and with a control group composed of 20 unaffected age-matched individuals. The 3D gait analysis was used to assess kinematic data and spatio-temporal parameters. Compared to left hemiplegic patients, right hemiplegic patients showed worse spatio-temporal parameters (*p* < 0.05) and better kinematic parameters (*p* < 0.05). Both pathological groups were characterized by abnormal gait parameters in comparison with the control group (*p* < 0.05). These findings show an association between the side of the lesion—right or left—and the different stroke patients’ gait patterns: left hemiplegic patients show better spatio-temporal parameters, whereas right hemiplegic patients show better segmentary motor performances. Therefore, further studies may develop and assess new personalized rehabilitation strategies considering the injured hemisphere and brain asymmetry.

## 1. Introduction

Stroke is one of the leading causes of disability-adjusted life years [1]. It is estimated that in 2030 the number of stroke deaths will reach 12 million, while the number of survivors will reach 70 million [2]. Among the different sequelae, gait impairment is one of the most debilitating: more than 80% of the survivors experience walking disabilities and 50–60% continue to have some impairments despite rehabilitation [3,4,5]; more specifically, 35% do not regain useful gait function, and 20–25% require full physical assistance to walk [6].

Important information to improve recovery may be found in the brain asymmetry (i.e., the specialization of the two hemispheres); in fact, looking at the role of the hemispheres and gait, the right hemisphere is related with impedance, spatial orientation, the static part of the movement and the position control, while the left hemisphere is related with the movement dynamics, motor control and trajectory [7,8,9]. This lateralization connected with motor performance has been highlighted in recent studies with stroke survivors, which showed that poor intersegmental coordination, direction and linearity of reaching movements are associated with a lesion in the left hemisphere [10,11,12], while a reduced capacity to shift the body weight between the legs, and poor body vertical orientation, body sway and stance control are associated with a lesion in the right hemisphere [7,13]. 

Considering these differences, hemiparetic patients may have a different recovery depending on the side of the lesion, as shown in a cross-sectional study where patients with left hemiplegia had a faster improvement in the functional ambulation profile and the velocity compared to patients with right hemiplegia [14], followed by an observational study based on a retrospective chart review where patients had changes in the symmetry values and in the centre of pressure depending on the side of the lesion [15]; then, specifically looking at the right lesion, a recent trial assessed if stroke survivors with a right hemisphere injury showed poorer improvements in balance, and it found that they made more mistakes compared to patients with left injury when performing more complex tasks [16]. Lastly, it was also assessed that patients with a right lesion had a marginally higher minimal detectable change (MDC) in weight-bearing asymmetry compared to patients with a left lesion (7.3% and 5.4%, respectively), while the velocity of sway was 13 mm/s in patients with left lesions and 9 mm/s in patients with right lesions [17]. These findings suggest that it is necessary to perform different treatment methods and strategies that take into account the side of the stroke. However, there is still a paucity of studies investigating the side of the lesion and its influence on gait, which would, thus, provide the implementation of new interventions and a better quality of life for stroke survivors. In fact, most of the literature about gait in stroke individuals is focused on the quantification of functional limitation during walking with no investigation in terms of the side of the lesion [18,19,20,21,22]. 

This retrospective study aims to bring further knowledge about the difference between left and right hemiplegia related to the side of the lesion and to understand whether the injured side, and therefore the predominance of the other hemisphere, can influence the clinical characteristics of the hemiplegic patient’s gait. This understanding could guide therapeutic choices and the characteristics of the rehabilitation program. 

## 2. Materials and Methods

### 2.1. Participants

For this retrospective cohort study, the data came from all the hemiplegic patients who had an instrumented gait analysis during the years 2004–2018 at the San Giuseppe Hospital, Istituto Auxologico Italiano, Piancavallo, Verbania, Italy. If patients had attended more than one visit at the clinic during this period, only the data from their first visit were considered. Inclusion criteria were: age > 18 years, presence of paresis in one lower limb, ability to understand instructions for performing the gait analysis test and ability to walk 10 m without the assistance of another person or walking aids. Exclusion criteria were: patients with bilateral stroke, those with a history of other neurological or musculoskeletal disorders unrelated to stroke, and left-handed.

According to these criteria, the study had a final sample size of 33 post-stroke patients (17 left and 16 right hemiplegic patients) who had the following characteristics: all patients were classified according to the classification of Bamford [23], and had characteristics of partial anterior circulation infarcts (PACI) assessed by CT scan at the hospital and a clinical functional evaluation, showing hemiparesis and similar functional motor skills and strength measured with Berg Balance Scale (BBS), Trunk Impairment Scale (TIS), Functional Ambulation Category (FAC), the Ten-Meter Walk Test (10MWT), motricity index and Functional Independence Measure (FIM). All the clinical evaluation values show similar results considering patients with nearly identical functional characteristics except on the side of the lesion.

On the other hand, a group of 20 unaffected age-matched individuals was recruited from among the hospital staff as the control group for the computation of gait profile score (GPS) normal values. Exclusion criteria for the control group were the existence of cardiorespiratory, neurological or musculoskeletal disorders. All of them showed normal flexibility and muscle strength, no evident gait abnormality and were able to walk independently. 

The study was performed in accordance with the ethics standards of the Institute Auxologico Italiano with approval of the study by the ethics committee on 20th July 2021 and with the Declaration of Helsinki 1964 and its latest amendments. Written informed consent was obtained from all participants. 

### 2.2. Experimental Setup 

All participants were evaluated with 3D gait analysis (3D-GA) at the Movement Analysis Lab of the San Giuseppe Hospital, Istituto Auxologico Italiano, Piancavallo (VB), Italy, using an optoelectronic system composed of six cameras (VICON, Oxford Metrics Ltd., Oxford, UK) set at 100 Hz, and two force platforms (Kistler, Winterthur, CH; acquisition frequency: 500 Hz). To evaluate the kinematics of each body segment, passive markers were positioned on the participants’ body, as described by Davis III et al. [24], and the underlying skeletal model was scaled on behalf of anthropometric data (height, weight, tibial length, distance between the femoral condyles or diameter of the knee, distance between the malleoli or diameter of the ankle, distance between the anterior iliac spines and thickness of the pelvis). After placing the markers, the participants were asked to walk barefoot at self-selected speed along a walkway where the force platforms were embedded in its central part. Kinematic and kinetic data were collected for each individual from at least five trials to guarantee the reproducibility of the results. For each participant (both patients and controls), three trials consistent in terms of gait pattern (spatio-temporal, kinematic and kinetic) were considered for the analysis.

### 2.3. Data Analysis

The related positions of each joint and joint centre were estimated through the motion analysis and human anthropometric data. The limb rotation algorithm is based on the determination of Euler angles with a y–x–z axis rotation sequence. The joint rotation angles that are routinely obtained correspond to flexion/extension, adduction/abduction and internal/external rotation, respectively. Therefore, the joint rotation angles that are clinically determined are trunk and pelvic obliquity–tilt–rotation, hip ad/abduction–flexion/extension–rotation, knee flexion/extension, ankle plantar/dorsiflexion and foot rotation. The trunk and pelvic angles are absolute angles, referenced to the initially fixed laboratory coordinate system; the hip, knee and ankle angles are all relative angles, e.g., the three hip angles describe the orientation of the thigh with respect to the pelvis; the foot rotation angle is an absolute angle, referenced to the laboratory, which indicates the position of the subject’s foot with respect to the direction of progression [24]. Kinematic data obtained from 3D-GA were normalized as a percentage of gait cycle, thus providing the trends of joint angle for pelvis, hip, knee and ankle, using specific software (Polygon Application, Oxford Industrial Park, Yarnton, UK; version 3.5.2.). They were also processed to compute the GPS, a parameter that summarizes the overall deviation of kinematic gait data relative to unaffected population [25]. Such an approach was implemented in this study as described by Baker et al. [25], and using SmartAnalyser software (BTSBioengineering, Milan, Italy; version 1.10.465). From a mathematical point of view, the GPS represents the root mean square (RMS) difference between the individual’s joint curve and the average curve calculated for a reference population of unaffected individuals. The overall GPS is based upon 15 clinically important kinematic variables (pelvic tilt, obliquity and rotation, hip flexion, abduction and internal rotation, knee flexion, dorsiflexion and foot progression for left and right sides), which are expressed as gait variable scores (GVSs), each of which represents the RMS difference between a specific time normalized gait variable and the mean data of a population of healthy individuals. The GPS is the RMS average of the GVS variables Equation (1)

(1) $$\mathrm{GPS}=\sqrt{\sum_{i=1}^{N}GVS_{i}^{2}}$$

In this analysis, a GPS score for each side was used based on all nine GVSs for that side. As the GPS represents the difference between the patient’s data and the average from the reference dataset, the higher the GPS value is, the lower the physiological gait pattern. GPS values for unaffected individuals lie in the range 5–6° [26,27,28]. The main spatio-temporal parameters (gait speed, step length, cadence and stance duration) were calculated; as for step length, the normalised step length (normalised respect to individual’s height) was reported. In this study, the kinetic data were not analysed, even if acquired.

### 2.4. Statistical Analysis

Statistical analysis was carried out using the Statistica (version 7.0; StatSoft Inc., Tulsa, OK, USA) software. All the parameters were computed bilaterally for each participant. All the parameters of interest resulted normally distributed (after the Shapiro–Wilk test), and a parametric statistic was used. One-way ANOVA was used to compare the paretic lower limb with non-paretic lower limb for right and for left hemiplegic patients and control group. Furthermore, post hoc analysis was performed to assess the contribution of each group in the variance of the spatio-temporal parameters and GPS (and its GVSs). Then, data of the right and left hemiplegic groups were compared using *t*-test for independent samples, in order to detect significant differences between the two groups. The level of significance was set at *p* < 0.05.

Assuming a significance level of 0.05 and using a t-test applied to the outcome variable for comparison (gait speed), it was verified that with the available dataset it was possible to obtain a level of statistical power of 0.73. This calculation was performed using GPower software. The effect size was computed for paretic and non-paretic size of the two groups; according to Cohen criteria, the effect size is considered small when ≤0.3, moderate when 0.4–0.7 and large when ≥0.8.

## 3. Results

A total of 63 hemiplegic patients were screened for eligibility, and 30 were excluded either because their lesion was in a different area, thus not corresponding to PACI, or because they had different functional characteristics, such as motor skills and strength, when they had been evaluated with the clinical functional evaluation mentioned above. Therefore, 33 hemiplegic patients (17 left and 16 right hemiplegic patients; mean age: 58.24 years; time since stroke event 3.66 years) fulfilled the eligibility criteria and were included in the study. For the control group, all the 20 unaffected age-matched individuals (mean age: 53.9 years) were included. Anthropometric and clinical features are reported in Table 1.

In Table 2, the spatio-temporal parameters and GPS (and its GVSs) values are reported for the paretic and non-paretic lower limbs of the right and left hemiparetic groups.

Data related to the spatio-temporal parameters showed that both pathological groups were characterised by abnormal gait parameters in comparison with the control group. In particular, the right hemiparetic group displayed lower gait speed (*p* = 0.009; Cohen’s d = −0.72) and cadence (*p* = 0.002; Cohen’s d = −0.69) and longer stance duration of the paretic side (*p* = 0.048; Cohen’s d = −0.33) compared to the left hemiparetic group.

As for kinematic data, the GPS values and most of the GVSs were statistically different with respect to the CG in both pathological groups. The GPS values for paretic and non-paretic lower limbs were similar from a statistical point of view (*p* > 0.05) in the comparison between the right and left hemiparetic group. As for GVSs, the right hemiparetic group presented lower values in terms of pelvic rotation (*p* = 0.011; Cohen’s d = −0.96), hip rotation (*p* = 0.046; Cohen’s d = −0.76 for the non-paretic side) and for foot progression (*p* = 0.029; Cohen’s d = −0.87 for the paretic side) in comparison with the left hemiparetic group. No other significant differences were observed.

## 4. Discussion

This study shows that the gait patterns of chronic post-stroke patients changed depending on the hemiplegic side, showing different and interesting walking adaptation strategies. As a matter of fact, patients with left hemiplegia are characterized by better spatio-temporal parameters (i.e., cadence, % stance and speed), thus showing superior walking velocity and adaptation concerning the quantity of movement; however, when looking at the function, they have worse cinematic parameters, with less physiologic walking ability in both the pathologic and healthy limbs. On the other hand, patients with right hemiplegia show worse spatio-temporal parameters but better GPS and GVSs; in fact, the values of the GVS related to the foot progression for the paretic side, the hip rotation for the non-paretic side and the pelvic rotation are lower. This occurs due to the reorganization of neuronal activity [29]: the damage in the right hemisphere leads to the prevalence of the left cerebral area, and consequently to better spatio-temporal parameters, whereas the damage in the left hemisphere leads to a prevalence of the right cerebral area, thus positively affecting the quality of movement. 

Our results for gait analysis agree with different studies, supporting their findings: a lesion in the left hemisphere is more correlated with poor intersegmental coordination, direction and linearity of movement [7,9,13], while a lesion in the right hemisphere affects the body’s vertical orientation, the stance control, the body sway, and the ability to shift the body weight between the legs [7,13]. Moreover, our results also agree with the findings by Kim et al. [14] where patients with left hemiplegia improved faster for the functional ambulation profile and the velocity compared to patients with right hemiplegia. Lastly, Ioffe et al. [9] also showed that the left hemisphere is connected with the dynamic characteristic and trajectory of the movement, while the right hemisphere is connected with its static part and the position control. Therefore, our results support all their findings since they show that left hemispheric damage leads to a reduced dynamic capacity of walking, whereas right hemispheric damage causes major problems regarding segmentary motor control. On the other hand, our results differ from those by Lopes et al. [8], who did not assess any significant difference between right and left hemiplegic patients. Then, many studies have highlighted that left hemiplegic patients have more severe impairments [30,31], which are often associated with hemispatial neglect disorder due to the neuroanatomy of the right hemisphere [32]. Our results partially agree: they correspond for quantity values but not for quality values; specifically, for the quality of movement. Hence, hemiparetic patients can have a different recovery depending on the side of the lesion, thus suggesting the implementation of different rehabilitation strategies.

An explanation to our findings can be found in the different neurofunctional characteristics of the hemispheres [33]: the left hemisphere regards speech, math calculations, analysis, logic and space–time relation, while the right hemisphere is associated with emotion and creativity; therefore, the two sides have substantial differences, thanks to which they complete each other, and, with the support of the callosal commissure, which allows interhemispheric communication, the hemispheres exchange motor, sensitive and cognitive information. Even if the role of the callosal commissure is not entirely understood, several studies have reported some important functions and characteristics: an interesting article concerning the neuroanatomical differences of Albert Einstein’s brain showed a different structure, which consisted of the different dimension of the callosal commissure [34]. An integration of and better use of the exchange between the hemispheres can play a fundamental role in the importance of the brain function. Interesting researches on split-brain patients clarified many fundamental aspects concerning the functioning of the hemispheres and the importance of the connection between the callosal commissure, and they also demonstrated the complexity of the brain function and of these connections [35]. In fact, there is not only a difference between the right and left areas, but there are also connections between inferior and superior areas that have to be taken into account, above all when considering creativity. The functional impairment of walking is also associated with an increase in the anisotropy in the damaged posterior callosal commissure, and its structural connectivity is associated with motor coordination influencing the gait after stroke [36]. The stroke in one hemisphere suddenly interrupts the balance and completeness by modifying the functions of both the contralateral and unilateral side with damage to the afferent flux from both sides of the body, influencing the whole cerebral functionality with different adaptive characteristics depending on the left or right hemisphere [37].

It seems that our results correspond to the neurofunctional characteristics of the hemispheres and the adjustments caused by stroke. With a left lesion, there is the loss of the rational part, thus affecting the dynamics and the rhythm of the movement, while with a right lesion there is the loss of the visuo-spatial part, thus affecting the control of the movement. These findings suggest new rehabilitation strategies need to performed that are more specific depending on the hemiplegic side. Specific stimuli directed towards the damaged area may improve the neuroanatomical connections between the hemispheres, bringing better motor, sensitive and cognitive adjustments. For instance, rehabilitative logic exercises, metaphors, the use of number sequences, Sudoku, crosswords, puzzles and time repetitions may stimulate the left lesion; on the other hand, outside orienteering exercises, poems, fables, tactile works, art therapy or music therapy may improve the right lesion. Concerning this, for example, it is interesting to underline how music stimulates the right hemisphere in non-musical people, while it stimulates the left hemisphere in professional musicians, showing how the functionality varies in time, experiences, learning phases, etc. These results can be applied to a new more personalized and specific rehabilitative strategy, but it may be that they can also be implemented in the rehabilitative–educational and development field. Evaluating the patients’ characteristics may help us to choose more analytical, temporal, verbal and calculation strategies for the left hemisphere, while choosing non-verbal, global and creative strategies for the right side. A rehabilitation stimulation, considering the cerebral asymmetry, can determine a more specific and effective response. Rehabilitation protocols capable of training and stimulating the injured area could lead us to new results in cognitive, functional and motor terms.

Despite our findings, it is important to consider some limitations, such as the sample size, which led to a not strong statistical power (0.73), and the kind of stroke. It is important to underline that according to the literature about the MDC of the gait profile score for post-stroke patients [38], the MDC values found for the GPS for the paretic lower limb (PLL) and non-paretic lower limb (NPLL) were 2.3° and 2.9°, respectively. Regarding the MDC for the GVS for the PLL and NPLL, the values were also very similar, with the exception of hip rotation (NPLL: 7.4°; PLL: 10°), knee flexion/extension (NPLL: 3.1°; PLL: 5.3°) and foot progression angle (NPLL: 4.1°; PLL: 6.2 ), for which we observed a larger MDC for the PLL. The significant results of the present study are globally in line with these data. In addition, ischaemic stroke (62.4%) is more prevalent than intracerebral haemorrhage (27.9%) and subarachnoid haemorrhage (9.7%) [39], and—regarding handedness—just 10% of the population is left-handed and 1% is ambidextrous [40]. Therefore, we think that our findings can be considered relevant and they could be generalizable, even if further studies with a larger sample should be conducted. As stated before, previous studies had similar results [7,9,10,11,12,13,14], supporting our findings. In addition, both males and females were pooled, not disaggregating by gender; combining male and female subjects, though, introduces a source of potential variability. However, with our sample it was not possible to consider them separately; thus, potential differences in movement characteristics between males and females need further study. Then, patients were selected based on their CT scan and a clinical functional evaluation; the magnetic resonance image would have provided more information concerning the lesion. Finally, although the patient was assessed with functional scales, an assessment of spasticity could have been included using the Ashworth scale.

Based on our results, in the future it may be interesting to invest in researches analysing the effects of the different kind of stimuli and treatments according to the cerebral lesions. It would lead to more effective rehabilitation protocols, which would further improve the stroke patients’ clinical conditions. 

## 5. Conclusions

The data show that ischaemic stroke patients have different gait patterns depending on the side of the lesion: left hemiplegic patients are characterized by better spatio-temporal parameters, whereas right hemiplegic patients show better segmentary motor performance and better GPS and GVSs values. This difference may be explained by the brain’s physiological asymmetry. Hence, we suggest the development of future studies assessing new personalized rehabilitation strategies to evaluate if a hemisphere-specific approach can further improve stroke patients’ clinical conditions.

## Figures and Tables

**Table 1:** Participants’ characteristics.

	Stroke (n = 33)	Control Group (n = 20)
Gender, n (%)		
Male	20 (60.6)	10 (50)
Age	58.24 ± 13.29	53.9 ± 11.2
Height (m)	1.68 ± 7.66	1.71 ± 8.23
Body mass (kg)	76.97 ± 17.15	68.9 ± 13.2
Time since stroke (years)	3.66 (1.84)	
Stroke type, n (%)		
Ischaemic	33 (100)	
Affected hemisphere, n (%)		
Right	16 (48.5)	
Left	17 (51.5)	

**Table 2:** Mean and standard deviation values of analysed parameters (spatio-temporal parameters, GPS and its GVSs) for the paretic and non-paretic side of the right and left hemiparetic group and the control group.

	Right Hemiplegia		Left Hemiplegia		Cohen’s d for Paretic Side	Cohen’s d for Non-Paretic Side	Control Group
	*Paretic Side*	*Non-Paretic Side*	*Paretic Side*	*Non-Paretic Side*			
		**Spatio-temporal parameters**					
Gait speed (m/s)	0.52 (0.13) *+		0.61 (0.12)+		−0.72		1.08 (0.17)
% stance (%gc)	64.94 (11.65)*+	70.99 (13.05)+	61.80 (6.60)+	69.26 (6.20)+	0.33	0.17	58.95 (1.85)
Step length	0.24 (0.07)+	0.22 (0.07)+	0.24 (0.07)+	0.24 (0.08)+	0	−0.27	0.59 (0.05)
Cadence (step/min)	76.23 (23.76) *+		90.80 (18.10)+		−0.69		103.00 (8.30)
		**GPS and GVSs (degrees)**					
GPS	10.19 (3.09)+	10.71 (3.58)+	10.74 (2.98)+	11.95 (3.53)+	−0.18	−0.35	7.61 (0.97)
Pel tilt	5.79 (3.85)		5.99 (4.09)		−0.05		5.12 (3.28)
Pel obl	4.01 (2.17)		4.89 (2.92)		−0.34		2.60 (0.93)
Pel rot	6.31 (3.03) *+		9.38 (3.35)+		−0.96		4.70 (1.43)
Hip flex	10.97 (5.18)+	11.56 (6.01)+	12.13 (5.62)+	11.88 (6.73)+	−0.21	−0.05	6.86 (3.50)
Hip abd	5.26 (3.25)	7.24 (6.19)	5.01 (2.50)	5.60 (3.30)	0.05	0.33	5.41 (2.76)
Hip rot	14.74 (7.88)+	12.99 (7.55) *+	12.24 (6.48)+	19.77 (10.05)+	−0.35	−0.76	8.35 (4.37)
Knee flex	14.78 (5.44)+	14.04 (6.03)+	15.47 (5.49)+	16.52 (5.01)+	−0.13	−0.45	7.87 (2.10)
Ank dorsi	11.59 (4.09)+	13.31 (5.63)+	11.17 (3.85)+	13.14 (3.60)+	0.11	0.04	5.63 (2.14)
Foot prog	6.00 (2.23) *	8.13 (4.58)	11.46 (5.15)+	6.59 (3.60)	−1.37	0.37	7.74 (3.98)

* *p* < 0.05, right vs. left hemiplegia. + *p* < 0.05, paretic/non-paretic side vs. healthy group. Abbreviations: gc: gait cycle; GPS: gait profile score; GVS: gait variable Score; Pel tilt: pelvic tilt; Pel obl: pelvic obliquity; Pel rot: pelvic rotation; Hip Flex: hip flexion: Hip abd: hip abduction; Hip rot: hip internal rotation; Knee flex: knee flexion; Ank dorsi: ankle dorsiflexion; Foot prog: foot progression.

## Data Availability

The datasets generated during and/or analysed during the current study are available in the Zenodo repository with the identifier https://doi.org/10.5281/zenodo.6548295. Accessed on 14 May 2022.
